# The gut microbiota composition is shaped by disease activity and individual treatment responses in patients with multiple sclerosis

**DOI:** 10.3389/fimmu.2025.1681342

**Published:** 2026-02-12

**Authors:** Veronika Ticha, Stepan Coufal, Zuzana Jiraskova Zakostelska, Tomas Thon, Radka Roubalova, Tomas Hrncir, Miloslav Kverka, Miluse Pavelcova, Pavlina Kleinova, Jana Lizrova Preiningerova, Ivana Kovarova, Jakub Kreisinger, Helena Tlaskalova-Hogenova, Eva Kubala Havrdova

**Affiliations:** 1 Department of Neurology and Centre of Clinical Neuroscience, First Faculty of Medicine, Charles University and General University Hospital in Prague, Prague, Czechia; 2 Laboratory of Cellular and Molecular Immunology, Institute of Microbiology of the Czech Academy of Sciences, Prague, Czechia; 3 Laboratory of Gnotobiology, Institute of Microbiology of the Czech Academy of Sciences, Novy Hradek, Czechia; 4 Laboratory of Animal Evolutionary Biology, Faculty of Science, Department of Zoology, Charles University, Prague, Czechia

**Keywords:** cladribine, disease modifying therapies, fingolimod, interferon-β, multiple sclerosis, neuroinflammation, serum biomarkers

## Abstract

Multiple sclerosis (MS) is a chronic autoimmune disorder of the central nervous system, with both animal and human studies highlighting a pivotal role for the gut microbiota in its pathogenesis. In this cross-sectional study, we investigated the potential role of gut microbiota in treatment response by analyzing its composition using 16S rRNA sequencing in treatment-naïve patients and those receiving disease-modifying therapies (interferon-beta (IFN-β), fingolimod, or cladribine), compared to healthy controls (HC). We also analyzed differences in gut microbiota composition and the serum levels of biomarkers associated with microbial translocation and inflammation based on treatment response. We found that individuals with clinically isolated syndrome (CIS) and treatment non-responders (NR) had significantly different alpha and beta diversity compared to HC. This effect was present in both IFN-β and fingolimod treatment. Individuals treated with cladribine had significantly different alpha and beta diversity regardless of the treatment outcome. The main differences in abundances in CIS and NR were found in bacteria that produce short-chain fatty acids. These patients also had significantly higher levels of lipopolysaccharide-binding protein and mannose-binding lectin compared to HC suggesting the compromised gut barrier function in multiple sclerosis leading to higher level of microbial translocation in these patients. In summary, we found that the treatment influences gut microbiota. Similar profile of gut microbiota and higher levels of molecules associated with microbial translocation were observed in patients with active disease (CIS and NR), suggesting the higher permeability of their gut barrier leading to pro-inflammatory tunning of their immune system.

## Graphical Abstract

**Figure:**
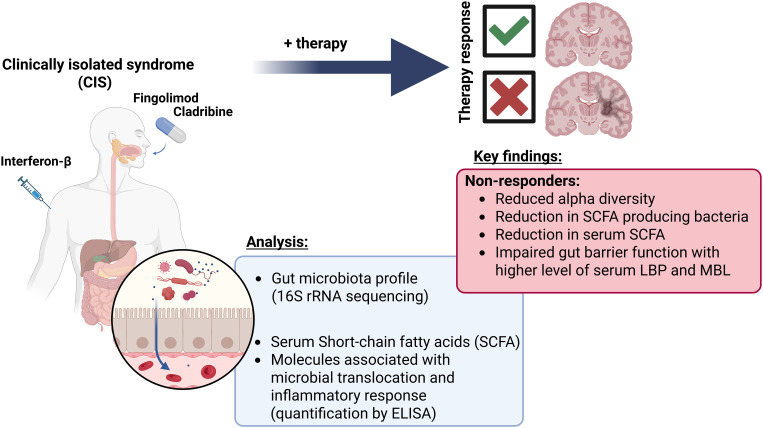
Created with BioRender.com.

## 1 Introduction

Multiple Sclerosis (MS) is a chronic inflammatory disease characterized by demyelination, axonal damage and neuronal loss driven by autoreactive T and B lymphocytes and macrophages (1). An altered composition of the gut microbiota - dysbiosis, has been observed in patients with both active and progressive MS, as well as in pediatric MS, regardless of treatment status (2–7). The presence of certain microbial taxa has been associated with an increased risk of relapse or MRI activity (7). Changes in gut microbiota composition can alter interactions with the gut-associated lymphoid tissue (GALT), promote intestinal inflammation and barrier disruption, and increase intestinal permeability (“leaky gut”), thereby facilitating translocation of microbes and their products and amplifying systemic immune activation. In this way, the microbiota can contribute to the initiation and propagation of autoimmune processes in distant organs such as the brain (8). Among the best-studied pathways, bacterial short-chain fatty acids (SCFAs) promote the differentiation and function of regulatory T cells (Tregs) and have been associated with improvement in MS disease course. A reduction in SCFA-producing bacteria belonging to Clostridia clusters IV and XIVa has been observed in persons with multiple sclerosis (PwMS), and this decrease may contribute to an imbalance between pro- and anti-inflammatory immune activity in these patients (9, 10). In addition, other microbiota-derived metabolites, including tryptophan metabolites, bile acids and phytoestrogens, are increasingly recognized as important modulators of CNS-directed immunity in MS, although these pathways were beyond the scope of the present work. Currently, there is no cure for MS. The goal of treatment is to prevent disease relapses and the progression of neurological deficits. The current treatment strategy for MS is to start the DMT as early as possible, once a diagnosis of MS or clinically isolated syndrome (CIS) as the first demyelinating event affecting the CNS has been established. First-line treatments of low to moderate efficacy include injectable IFN-β and glatiramer acetate, oral teriflunomide and dimethyl fumarate and selective sphingosin-1-phosphate (S1P) agonists such as ponesimod and ozanimod. Second-line or high-efficacy therapies (HET) represent an ever-growing group of drugs with different mechanisms of action in MS. These include drugs that block immune cell trafficking, such as natalizumab and the non-selective S1P agonist fingolimod (FIN), pulse immune-reconstitution therapy with cladribine (CLA) and alemtuzumab, or anti-CD20 therapy ocrelizumab and ofatumumab. HET is intended for escalation of treatment in MS patients who fail on first-line treatment or as an early option for treatment-naïve patients at high risk of rapid disease progression and poor prognosis (11).

Fingolimod acts as a non-selective S1P functional antagonist. By blocking the S1P signaling pathway, it prevents lymphocyte egress from the lymph nodes and migration to target organs. Another effect of natural S1P pathway blocking could be the stabilization of the gut barrier by reinforcing the intestinal barrier integrity (12).

Cladribine is an antimetabolite that induces an initial reduction in both T and B lymphocyte counts, followed by their repopulation and reconstitution of normal effector function of the immune system (13). Cladribine is also used for the treatment of celiac disease. It has been shown to have a positive effect on the gut immune system (14).

IFN-β was the first Disease Modifying Drug approved for MS treatment, but its mechanism of action remains not fully understood. In addition to decreasing the production of interferon gamma, it has a direct anti-proliferative effect on T cells and influences the secretion of regulatory cytokines (15). Endogenous IFN-β appears to promote an anti-inflammatory response in the gut, reduce inflammation by suppressing IL-1b production and inflammasome activity, and maintain the immune system homeostasis (16).

Although numerous studies have investigated patients on different MS treatments, no specific microbiome composition pattern has been consistently associated with particular DMT. Furthermore, it remains unknown, whether the treatment response correlates with distinct microbiome composition compared to treatment failure. In our study, we therefore combined 16S rRNA profiling with measurements of serum pro-inflammatory molecules and SCFA concentrations to link taxonomic shifts and systemic inflammatory tone to one key microbial metabolic axis. Therefore, the aims of our study were 1) to analyze the gut microbiome composition in individuals with CIS and individuals treated with different types of DMTs and compare them with healthy controls (HC); 2) to find out differences in gut microbiota based on the treatment response and 3) to complement these results with the analysis of serum biomarkers associated with microbial translocation and inflammation.

## 2 Methods

### 2.1 Study cohort

Patients and healthy controls (HC) were recruited from a single academic hospital MS center between November 2018 and December 2023. Inclusion criteria were diagnosis of relapsing-remitting multiple sclerosis (RRMS) or individuals with clinically isolated syndrome (CIS), self-identified as white, and age between 18 and 60 years. Exclusion criteria were other autoimmune disease, known cancer or other conditions (e.g., gut disorders, psychiatric and mental disorders) that could affect the gut microbiota. For healthy subjects, exclusion criteria also included a use of antibiotics within three months prior to sampling. All study participants provided written informed consent. This study was approved by the Ethics Committee of General University Hospital in Prague 15/19 Grant AZV VES 2020 VFN.

A total of 143 patients and 81 HC were enrolled, including 47 individuals with CIS and 96 patients with RRMS diagnosed according to the 2017 McDonald diagnostic criteria, who had received disease modifying therapy (DMT) for at least two years. RRMS patients were treated either with IFN-β (38 patients), FIN (37 patients) or CLA (21 patients). Neurological examination and Expanded Disability Status Scale (EDSS) assessment were performed at the first visit, at which time patients provided fasting blood and stool samples. Clinical data, including age, sex, disease duration and EDSS were collected (**Table 1**). All patients underwent a baseline magnetic resonance imaging (MRI) scan of the brain and cervical spine, with follow-up scans conducted annually to assess the number of new or expanding T2 lesions. A relapse was defined according to the 2017 McDonald diagnostic criteria. The number of relapses, progression in EDSS, and presence of new or enlarging white matter lesions on MRI within the two years prior to study entry were considered to assess NEDA-3 status (No Evidence of Disease Activity, i.e., no relapses, no new/enlarging white matter lesions on MRI, and stable EDSS). Patients who met the criteria for NEDA-3 were classified as treatment responders (R group), while those who did not meet these criteria were classified as treatment non-responders (NR group).

**Table 1:** Summary of anthropometric and clinical parameters from patients with multiple sclerosis and healthy controls.

Characteristic	HC (N = 81)	CIS cohort (N = 47)	IFN-β (N = 38)	Fingolimod (N = 37)	Cladribine (N = 21)
Sex woman/men	42/39	29/18	26/12	24/13	15/6
Age, years	39 (27.5, 44.5)	35 (30, 43)	42 (33, 47.3)	45 (39.5, 51.5) a, b	37 (32, 46.5)
BMI	24.42 (21.6, 28.8)	24.54 (21.9, 29.7)	25.35 (22.4, 28.4)	25.33 (23.9, 29.2)	24.98 (22, 28.7)
Age at onset	NA	35.5 (29, 41.3)	31.5 (27, 36)	29 (22.5, 34)	27 (24, 35)
Disease duration	NA	NA	6 (3, 12.5)	16.4 (10.7, 22.1)	9.5 (4.4, 13.9)
Responders /Non responders	NA	NA	16 (R-IFN)/22 (NR-IFN)	7 (R-FIN)/30 (NR- FIN)	8 (R-CLA)/13 (NR-CLA)
EDSS	NA	2 (1.5, 2.5)	2 (1.5, 2.5)	2.5 (1.8, 3.5)	2 (2.0, 3.0)

Medians are reported with the first and third quartiles in parentheses. N, number of participants; BMI, body mass index; EDSS, Expanded Disability Status Scale; NA, not applicable. Statistical differences between groups are depicted by letters at the level of p < 0.05. When the letter a is present in one row of the table, it means there is statistically significant difference between the marked group and healthy controls. When the letter b is present in one row of the table, it means there is statistically significant difference between the marked group and CIS group (R – responder, NR – non-responder).

Stool and blood serum samples from healthy controls and both individuals with CIS and RRMS patients on DMTs were collected as previously described (17). All samples were stored at −80°C until processing.

### 2.2 Serum biomarkers analysis

Biomarkers related to gut barrier function and inflammatory response or produced in response to microbial translocation were analyzed and quantified in serum using a commercial enzyme-linked immunosorbent assay (ELISA) (**Table 2**).

**Table 2:** The list of quantified biomarkers in sera.

Biomarker	Abbreviation	Manufacturer	Cat. no.
Mannan-Binding Lectin	MBL	R&D systems	DY2307
Osteopontin	OPN	R&D systems	DY1433
Lipopolysaccharide-Binding Protein	LBP	R&D systems	DY870
Soluble CD14	CD14	R&D systems	DY383
S100A8/S100A9 heterodimer	S100A8/S100A9; calprotectin	R&D systems	DY8226-05
Interleukin-18	IL-18	R&D systems	DY318-05
Lipocalin-2/NGAL	Lipocalin	R&D systems	DY1757
Serum Amyloid A	SAA	R&D systems	DY3019-05
Human Short-Chain Fatty Acids (ScFA)	SCFA	Mybiosource	MBS7269061

### 2.3 Gut microbiota analysis

The gut microbiota was analyzed as previously described (17). Briefly, DNA was isolated from the stool samples using the ZymoBIOMICS DNA Miniprep Kit (Zymo Research, Irvine, CA, USA) according to the manufacturer’s protocol. The V3 and V4 regions of the bacterial 16S rRNA gene were amplified by specific primers (341F GTCCTACGGGNGGCWGCAG and 806R GGACTACHVGGGTWTCTAAT) using the KAPA HiFi HotStart Ready Mix (Roche, Penzberg, Germany), as follows: initial denaturation step 3 min at 95°C followed by 25 cycles at 95°C for 30 s, 55°C for 30 s, 72°C for 30 s with a final elongation step at 72°C for 5 min. PCR products were checked with QIAxcel advanced capillary electrophoresis (QIAgen, Hilden, Germany). Triplicates of the amplicons were pooled and normalized with the SequalPrep™ Normalization Plate Kit (Thermo Fisher Scientific, Waltham, MA, USA), concentrated (Eppendorf centrifugal vacuum concentrator), and purified with DNA Clean & Concentrator Kit (Zymo Research). PCR amplification negative controls, extraction and sequencing positive controls (mock communities; ZymoBIOMICS Microbial Community Standard in linear and logarithmic form (MOCK 6300 and MOCK 6310 Zymo Research, USA) were processed in a similar manner. The amplicon libraries were then ligated with sequencing adapters using the KAPA HyperPlus Kit (Roche), pooled in equimolar concentrations, and sequenced 2x300 bp paired-end read using the MiSeq platform (Illumina, San Diego, CA, USA).

### 2.4 Sequencing data processing and statistics

In the first step, sample demultiplexing, primer detection, and trimming were performed using Skewer (18). Low-quality reads (expected error rate per paired-end read > 2) were then eliminated. DADA2 was used to denoise the quality-filtered reads and quantify 16S rRNA Amplicon Sequence Variants (ASVs) in each sample (19). Chimeric ASVs were detected and eliminated using UCHIME and the gold.fna reference database (https://drive5.com/uchime/gold.fa) (20). Taxonomical assignment of non-chimeric ASVs was conducted using the RDP classifier with an 80% confidence threshold and the Silva database (release 138) as a reference (21). Chloroplasts and mitochondria, as well as sequences that could not be assigned to a bacterial phylum, were considered contaminants of the diet or sequencing artifacts and were excluded from all downstream analyses. Sequences from technical duplicates were merged for each sample. The ASV abundance matrix (i.e., the number of ASV reads in each sample), ASV sequences, their taxonomic annotations, and phylogeny were merged into a single database along with sample metadata using the phyloseq package in R (R Core Team 2020) (22). To standardize sequencing depth, we rarefied the ASV table for alpha and beta diversity analysis. Rarefaction depth was set to the sample size of the minimal sequencing depth, i.e., where the majority of species were observed within a given number of samples. The alpha diversity was expressed as the Shannon diversity index, observed ASVs, and Faith’s Phylogenetic Diversity and compared using linear regression model adjusted for gender as additional factor. Principal Coordinate Analysis (PCoA) was performed to investigate differences in microbiota composition between samples. We used relative ASV abundance (Bray-Curtis index) and UniFrac (23–26). To evaluate the differences in beta diversity between groups, we used the Permutational Multivariate Analysis of Variance Using Distance Matrices (PERMANOVA) with a vegan package (27). Taxonomical analysis was performed using the microViz package, and relative abundances were calculated and presented (28). Associations between clinical metadata and microbial features were tested in R using the MaAsLin2 package after TSS normalization and LOG transformation of data (29). The ggplot2 package was used for graphical visualization of the data (30). Microbiome analysis sequencing data are available in the European Nucleotide Archive (ENA) (https://www.ebi.ac.uk/ena/browser/home) under accession number PRJEB85230.

The differences in serum biomarker levels between groups were tested using non-parametric Kruskal-Wallis test with Dunn’s multiple comparisons in GraphPad Prism statistical software (version 10, GraphPad Software, San Diego, CA, USA).

## 3 Results

### 3.1 Dysbiosis of the gut microbiota is a typical feature of both individuals with CIS and RRMS patients

**Figure 1:**
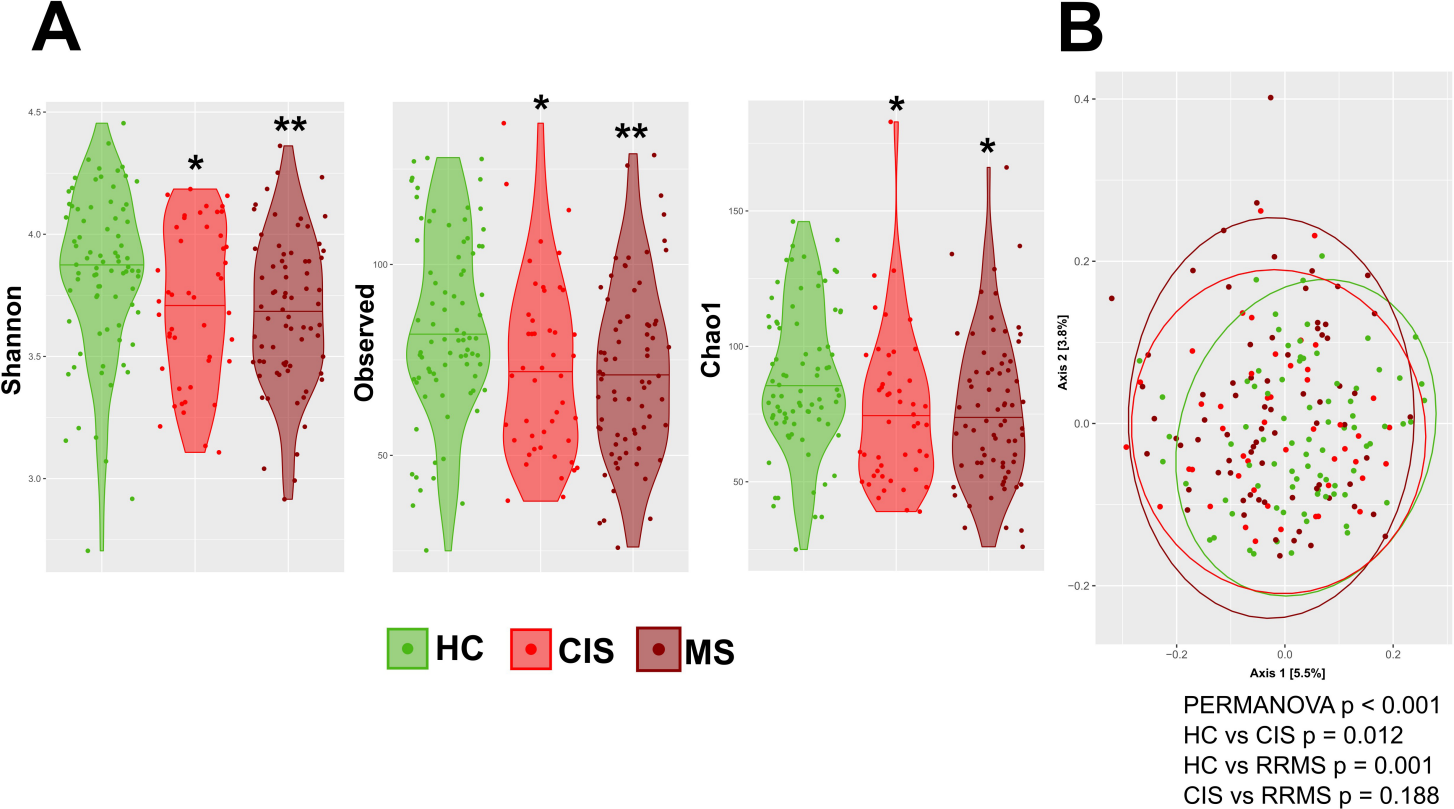
Composition of gut microbiota in newly diagnosed CIS and RRMS patients. **(A)** Alpha diversity was assessed using the Shannon diversity index, Observed ASVs, Faith’s PD metric. Significant differences among groups were tested using linear regression model adjusted for gender. **(B)** Principal Coordinate Analysis (PCoA) of beta diversity based on Bray-Curtis dissimilarity metrics of HC, and CIS, RRMS patients. Each point represents one sample and statistical significance was tested with PERMANOVA. (*p<0.05, **p<0.01, ***p<0.001).

We first compared the gut microbiota of individuals with CIS, patients with longer history of MS treatment (RRMS) and HC. We found that CIS and RRMS had significantly lower alpha diversity compared to HC, regardless of their treatment history (**Figure 1A**). And while both groups show significant changes in gut microbiota composition, beta diversity is similar in both groups of MS patients (**Figure 1B**).

### 3.2 Individuals with low alpha diversity do not respond to therapy

**Figure 2:**
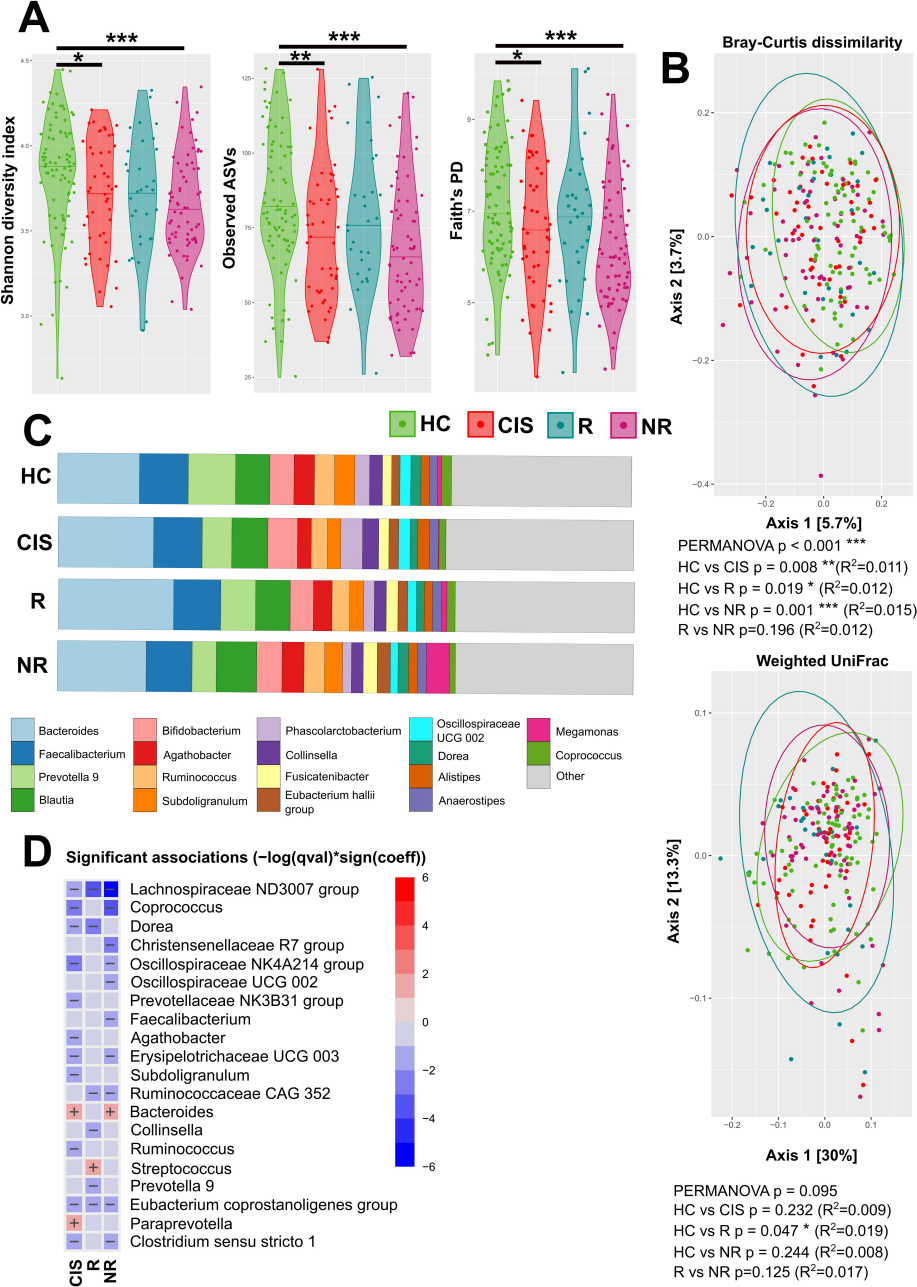
Gut microbial composition according the health status and treatment outcome **(A)** Alpha diversity was assessed using the Shannon diversity index, Observed spASVs, Faith’s PD metric. The significant differences among groups were tested using the linear regression model adjusted for gender. **(B)** Principal Coordinate Analysis (PCoA) of beta diversity based on Bray-Curtis dissimilarity metrics and weighted UniFrac, where each point represents patient or healthy control sample, tested with PERMANOVA. **(C)** Relative abundances of the top 18 most abundant taxa of gut microbiota at the genus level are presented as bar plots for each group. **(D)** The statistical differences in abundances of genera between the groups and HC were performed using the MaAsLin2 R package. (*p<0.05, **p<0.01, ***p<0.001).

When MS patients were divided into responders and non-responders, the significant decrease in alpha diversity was present only in non-responders (**Figure 2A**). But the gut microbiota beta diversity of either R and NR was significantly different from HC, as measured by the Bray-Curtis dissimilarity index (**Figure 2B**). There were no significant differences in beta diversity between responders and non-responders suggesting that while responders regain microbiome richness the disease signature in beta diversity is still present. The differences between R and NR are even documented on the relative abundances of the 18 most abundant taxa **Figure 2C**). When compared to HC with differential abundance analysis, each group has several specificities. In non-responders, four functionally protective microbial guilds are markedly depleted (i) core SCFA–producing bacteria (e.g. *Coprococcus* spp.*, Faecalibacterium* spp.*, Lachnospiraceae* ND3007 group*, Ruminococcaceae CAG-352, Oscillospiraceae UCG-002* group Chybí závorka. (ii) bacteria contributing to overall richness and ecosystem stability (e.g., *Christensenellaceae R7* group, *Oscillospiraceae NK4A214* group), (iii) cholesterol-to-coprostanol and secondary-bile-acid converters (e.g. *Ruminococcaceae CAG 352* group), and (iv) low-endotoxin Firmicutes (e.g. *Erysipelotrichaceae UCG003*) while the pathobiont genus *Bacteroides* expands. Many of bacteria significantly decreased in non-responders were found decreased also in CIS when compared to HC. *Christensenellaceae R7* group was able to distinguish NR from newly CIS with AUC = 0.612 (p=0.04). Relative to healthy controls, responders displayed a distinct microbiota signature characterized by a significant decrease of mucin-degrading bacteria (e.g. genera *Dorea*, *Collinsella* and *Prevotella 9*), together with increased abundance of genus Streptococcus, which is capable of producing γ-aminobutyric acid (**Figure 2D**).

### 3.3 The difference in dysbiosis between R and NR depends on their treatment

**Figure 3:**
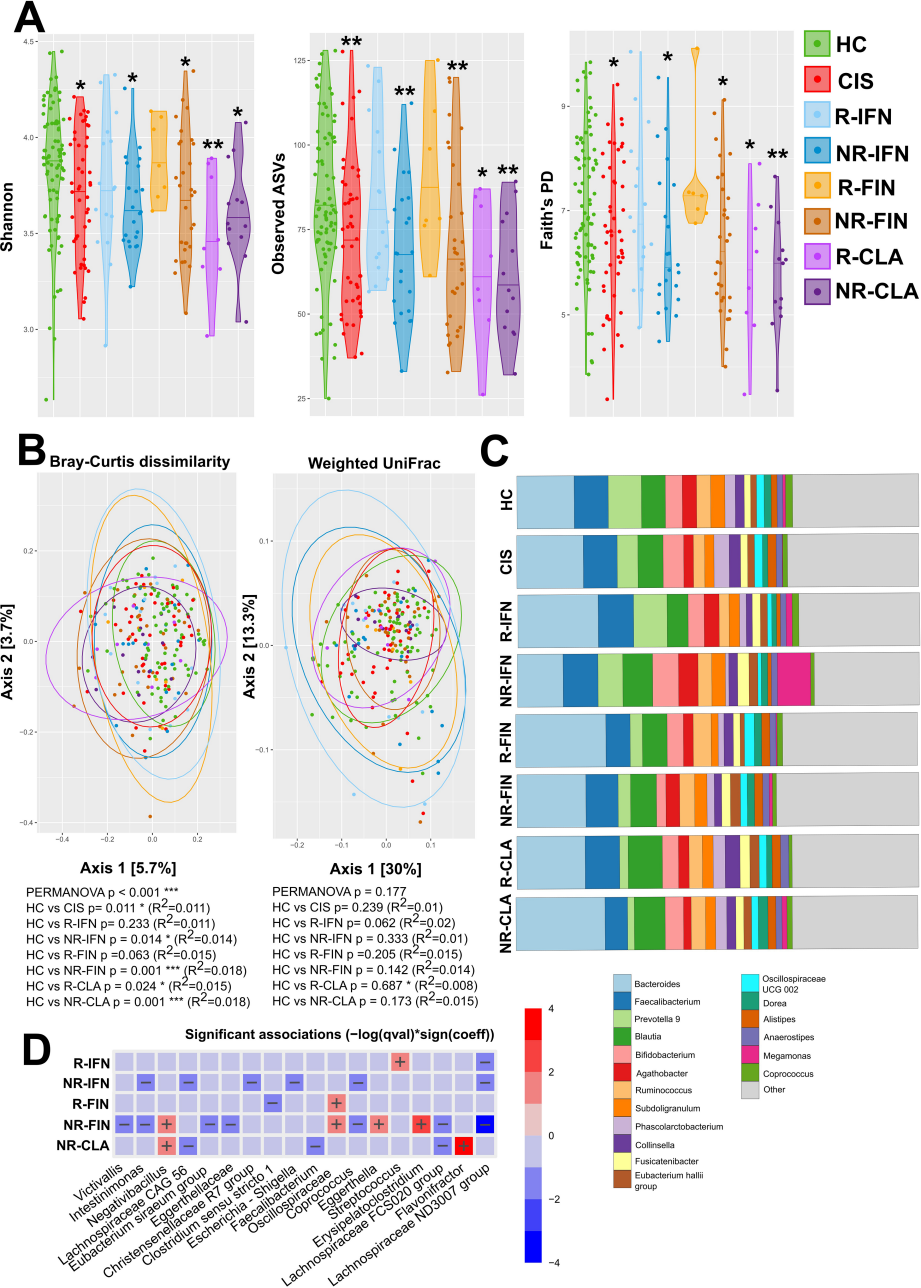
The composition of the microbiota is influenced more by the response to the therapy than by the medication. **(A)** Alpha diversity was assessed using the Shannon diversity index, Observed ASVs and Faith’s PD metric. The significant differences among groups were tested using the linear regression model adjusted for gender. *p<0.05, **p<0.01, ***p<0.001 compared to HC. **(B)** Beta diversity was evaluated using Bray-Curtis dissimilarities, weighted UniFrac and Principal Coordinate Analysis (PCoA) where each point represents patient or healthy control sample, tested with PERMANOVA. **(C)** Relative abundances of the top 18 most abundant taxa of gut microbiota at the genus level are presented as bar plots for each group. **(D)** The statistical differences in abundances of genera between the groups were performed using the MaAsLin2 R package. Statistical significance compared to HC is depicted by + (increased abundance) and – (decreased abundances).

Next, we analyzed which MS treatment failure, IFN-β, FIN or CLA, is preceded with dysbiosis by comparing the gut microbiota composition between R and NR. We found that while alpha diversity is significantly decreased in NR to all treatments. It is similar to HC in responders to IFN-β (R-IFN) and fingolimod (R-FIN), but not in responders to CLA (R-CLA) (**Figure 3A**). This suggests that while dysbiosis may influence the therapeutic success in IFN or FIN, gut microbiota dysbiosis does not prevent CLA from therapeutic success. Similar situation is documented on beta diversity, when Bray-Curtis dissimilarity index differs from HC in all study groups except the responders to IFN-β and FIN (**Figure 3B**). At the taxonomic level, differences were already evident when the relative abundance profiles for the 18 most abundant taxa were plotted (**Figure 3C**, **Supplementary Figure 1**). When compared to HC, we found that R-IFN higher levels of *Streptococcus* spp. The relative abundance of *Streptococcus* spp. and *Clostridium sensu stricto 1* was capable to distinguish R-IFN from NR-IFN with AUC = 0.768 (p=0.0047) and AUC = 0.696 (p=0.037), respectively. NR-IFN had lower levels of bacterial taxa Lachnospiraceae *ND3007* group, *Coprococcus*, *Escherichia-Shigella*, Christensenellaceae R7 group, Lachnospiraceae CAG 56, *Intestinimonas* compared to HC (**Figure 3D**). R-FIN had significantly lower level of genus *Clostridium sensu stricto 1* than HC. The *Oscillibacter* spp. was able to distinguish R-FIN from NR-FIN with AUC 0.719 (p=0.0034). NR-FIN had significantly lower levels of bacterial taxa Lachnospiraceae ND3007 group, Lachnospiraceae FCS020 group, *Coprococcus*, Eggerthellaceae*, Eubacterium siraeum group, Intestinimonas, Victivallis* and higher levels of *Erysipelatoclostridium, Eggerthella*, and *Negativibacillus* as compared to HC. Non-responders to cladribine (NR-CLA) had lower levels of Lachnospiraceae FCS020, *Faecalibacterium* spp., Lachnospiraceae CAG 56 and higher levels of genera *Flavonifractor* and *Negativibacillus*. Next to these findings, some taxa were enriched in both responders and non-responders within the same treatment arm (e.g. Oscillospiraceae in FIN; Lachnospiraceae ND3007 group in IFN), suggesting that some microbial shifts are driven by treatment exposure per se rather than by clinical response.

### 3.4 Individuals with clinically isolated syndrome and treatment NR demonstrated increased levels of MBL and LBP

**Figure 4:**
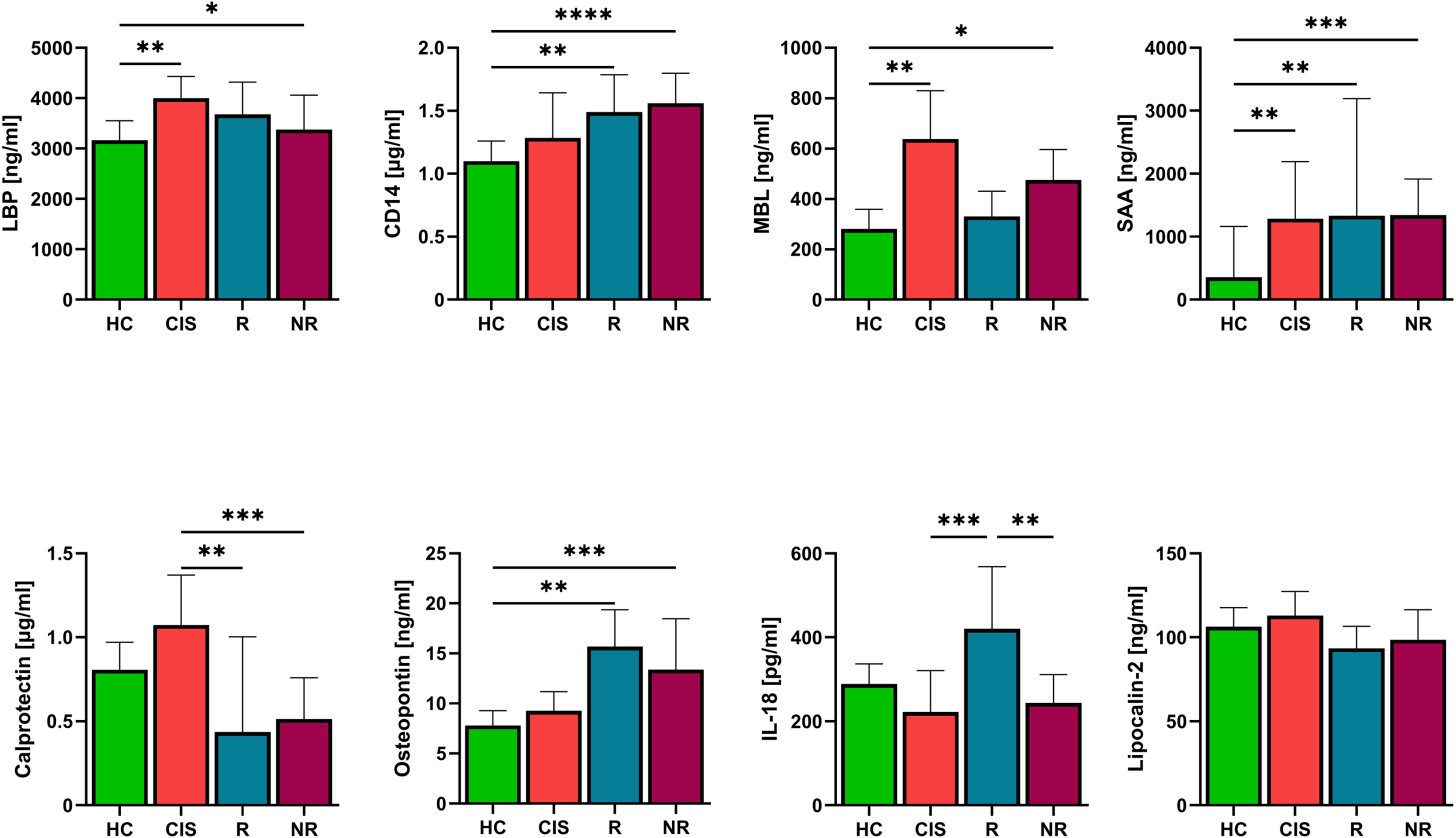
The levels of serum LBP, CD14, MBL, calprotectin, osteopontin and IL-18 in patients with CIS and RRMS patients according treatment response. Lipopolysaccharide-binding protein (LBP), soluble CD14 (CD14), Mannose-binding lectin (MBL), calprotectin, osteopontin, interleukin-18 (IL-18), Lipocalin-2, serum amyloid A (SAA). Statistical differences between groups were tested using the Kruskal-Wallis test with Dunn’s multiple comparisons *p<0.05, **p<0.01, ***p<0.001, ****p<0.0001 (HC n=63, CIS n=35, R n=30, NR n=42).

Increasing evidence suggests that there is intestinal barrier dysfunction in MS. This could lead to microbial translocation and an altered neuroinflammatory response. For this reason, we measured molecules associated with microbial translocation (Lipopolysaccharide-binding protein (LBP), soluble CD14 (CD14), Mannose-binding lectin (MBL)) and inflammation (Calprotectin, Osteopontin (OPN), IL-18, Lipocalin-2, Serum Amyloid A (SAA)). The individuals with CIS, had significantly higher levels of MBL and LBP, compared to HC and were able to distinguish them from HC with AUC = 0.726 (p=0.0002) and AUC = 0.710 (p=0.0007), respectively. (**Figure 4**). NR had significantly higher levels of LBP, CD14, MBL and OPN compared to HC. CD14 and OPN were, however, significantly elevated also in responders as compared to HC (**Figure 4**). Compared to patients receiving long-term treatment, the CIS patients had significantly higher calprotectin levels (**Figure 4**). In responders, we found significantly higher levels of IL-18 compared to active disease (CIS, NR) (**Figure 4**). The level of acute-phase apolipoprotein SAA was significantly higher in individuals with CIS, R and NR group as compared to HC, suggesting low-grade systemic inflammation irrespective of the treatment response status. In contrast, serum Lipocalin-2 concentrations did not differ significantly between HC, individuals with CIS, R or NR group (**Figure 4**). If we then focus on the individual therapies, patients with IFN-β treatment had higher levels of LBP and CD14 compared to HC regardless of response (**Supplementary Figure 2**). NR-IFN had higher levels of MBL compared to HC. Treatment with CLA or FIN did not lead toany changes in the levels of LBP, CD14 and MBL (**Supplementary Figure 1**). Interestingly, NR-FIN had significantly lower level of calprotectin and significantly higher level of OPN than HC. Patients with CLA treatment had significantly higher level of OPN than HC regardless of the treatment response (**Supplementary Figure 2**). The IL-18 was capable to distinguish R from NR with AUC = 0.697 (p=0.0046).When we used the level of IL-18 to distinguish responders and NR to individual DMTs, we found that IL-18 was able to distinguish treatment response in IFN-β (AUC = 0.810, p = 0.001), FIN (AUC = 0.781, p = 0.047), but had no power to distinguish treatment response in patients treated with CLA (AUC = 0.600, p = 0.56).

### 3.5 Lower serum short chain fatty acids in individuals with CIS and non-responders

**Figure 5:**
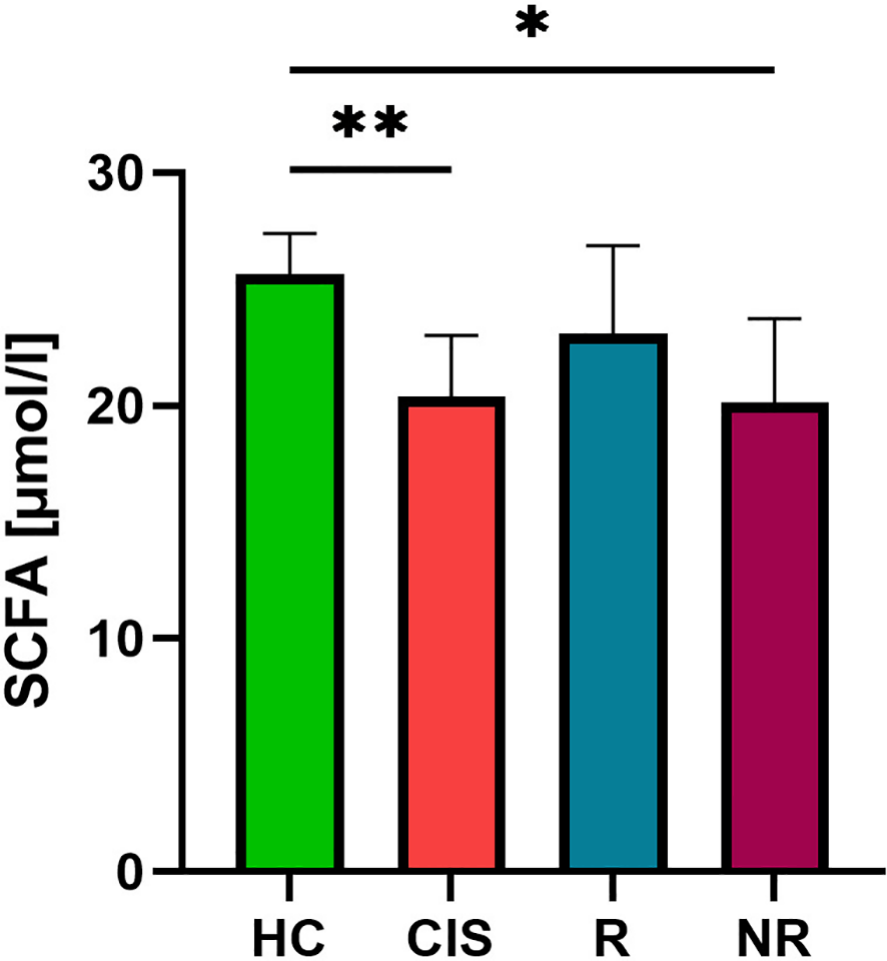
The levels of serum short chain fatty acids (SCFAs) in patients with CIS and RRMS patients according treatment response. Statistical differences between groups were tested using the Kruskal-Wallis test with Dunn’s multiple comparisons *p<0.05, **p<0.01 (HC n=22, CIS n=32, R n=25, NR n=42).

To assess the differences in abundances of SCFA producing bacteria we quantified short-chain fatty acids (SCFAs) directly in serum. Total serum SCFA concentrations were significantly reduced in individuals with CIS and NR compared with HC. We did not detect a statistically significant difference in serum SCFA levels between R and NR group; however, values in the R group were numerically closer to those of HC (**Figure 5**).

## 4 Discussion

In this study, we analyzed the gut microbiota aiming to find out the signatures associated with therapeutic success in MS. First, we found that patients with MS have decreased alpha diversity compared to healthy controls, regardless of if they were freshly identified (CIS) or suffer from MS for more than 2 years. This suggests that MS, and not MS treatment, induces dysbiosis. This is in agreement with multiple previous studies, which also observed a significant decrease in alpha and differences in beta diversity in MS patients compared to HC (31, 32). When taking the treatment status and treatment response into consideration, patients with CIS and NR had significantly lower alpha diversity compared to HC, as measured by Shannon diversity index, Observed taxa and Chao1 indices. Next to alpha diversity, we found significant differences in beta diversity between all study groups when compared to HC. An interesting and distinctive finding was observed when we stratified patients not only by treatment response (NEDA-3 status), but also by DMT type. Compared to HC, NR-IFN and NR-FIN showed significantly lower levels of alpha diversity. Both R-CLA and NR-CLA had decreased levels of alpha diversity. A similar pattern was seen in beta diversity, which was significantly different in NR to all three DMTs (NR-FIN, NR-IFN and NR-CLA), R-CLA and CIS patients compared to HC.

A recently published review identified 13 studies evaluating alpha and beta diversity in MS patients (33). Only five of these studies reported any significant differences. One cross-sectional study found significantly lower alpha diversity in patients with secondary progressive MS (SPMS) versus RRMS patients based on the Simpson diversity index, with a similar but not significant trend in Shannon’s diversity index (31). Another study using the Shannon’s diversity index reported significant reduction in alpha diversity in RRMS patients compared to HC (32). However, while no differences between total RRMS patients and HC were observed in another study, RRMS patients with active disease showed decreased species richness compared to those in remission and HC (34).

In our study, we demonstrated a significant difference in alpha and beta diversity between HC and patients with active MS (CIS and NR). However, no significant difference in alpha diversity was observed between patients with stable MS (R group) and HC. This observation was consistent regardless of the DMT used, except for patients treated with CLA, who showed significantly lower alpha diversity compared to HC.

Differences at the taxonomic level were primarily observed between patients with active MS (CIS and NR) and HC. We identified five bacterial taxa with lower abundance in both active MS groups compared to HC and one with higher abundance. Genus *Coprococcus*, *Erysipelotrichaceae* UCG003, *Eubacterium coprostanoligenes* group, *Clostridium* sensu stricto 1 and *Oscillospiraceae* of the NK4A214 group were reduced in both CIS and NR compared to HC, whereas *Bacteroides* were increased in these groups. This compositional shift is expected to lower butyrate availability, weaken epithelial tight-junction integrity, and raise systemic lipopolysaccharide exposure, collectively creating a pro-inflammatory milieu that diminishes the pharmacodynamic efficacy of MS disease-modifying therapies.

*Coprococcus* spp. is a butyrate-producing genus within the phylum Firmicutes, and its abundance is inversely correlated with several neuropsychological and neurodegenerative disorders, such as depression, language development disorder in children and Parkinson’s disease (35). A study investigating the role of SCFAs in MS found a dramatic depletion of bacterial genera belonging to the *Lachnospiraceae* family in MS patients compared to HC. Specifically, the proportions of the SCFAs producers *Roseburia* spp., *Coprococcus* spp., and *Blautia* spp. were reduced. Inverse correlations were also found between the levels of proinflammatory CD4+/IL-17+ T lymphocytes and the proportions of the SCFAs-producing genera, *Coprococcus* and *Ruminococcus*. Decreased abundance of *Coprococcus* spp., *Clostridium* spp., and an unidentified *Ruminococcaceae* family was observed in patients with active MS compared to HC along with increased CXCR3+ CD4+ and CD8+ T cells (32). These cell types were also inversely correlated with alpha diversity.

We observed a reduction in other SCFAs-producing bacteria and health-associated taxa in our patients with active MS, including genera *Clostridium* (in both CIS and NR), *Ruminococcus* and *Dorea* (only in the CIS) and *Faecalibacterium* and *Christensenellaceae* R7 group (only in the NR group). These species are known to be associated with lower severity in progressive MS (34). A significant reduction in *Christensenellaceae* R7 group and *Ruminococcaceae* family has also been reported in patients with pediatric MS, regardless of their treatment status, including DMT naïve and treatment exposed individuals (36).

An increased abundance of *Bacteroides* spp. was found in CIS and NR group. Similarly, higher levels of *Bacteroides* have been reported in patients treated with IFN-β and natalizumab compared to untreated patients. Conversely, decreased abundance of *Bacteroides* spp. was found in patients 6–12 months after initiating ocrelizumab treatment. This may reflect the dual immunomodulatory effects of the *Bacteroides* genus, which can be anti-inflammatory due to SCFAs production and induction of IL-10 secretion by Polysaccharide A (a component of the Bacteroides capsule), but also pro-inflammatory due to the overproduction and translocation of lipopolysaccharide (LPS) across the blood-brain barrier into the brain. LPS have also been detected in aged human brains around and within Alzheimer’s disease -affected neurons (37, 38).

Unlike our patients with active MS, patients with stable MS (R group) showed lower levels of genera *Collinsella*, and *Prevotella* 9 and higher levels of *Streptococcus* compared to HC. Higher abundance of *Collinsella* spp. has been previously reported to be associated with higher T2 lesion volume and higher EDSS (39). The decrease in *Prevotella* spp. abundance in MS patients has already been described with a statistically significant negative correlation demonstrated between the relative abundance of *Prevotella* spp. and the Th17 cell frequency in the human small intestine (40). In contrast to our findings, a study on relapsing-remitting MS patients with inactive disease reported higher abundance of *Prevotella* spp. not only in comparison to patients with active disease but also to the HC group. This study proposed a protective role for this beneficial, anti-inflammatory strain suggesting that it may counterbalance the effect of Th17 cell–inducing microbes in the pathogenesis of MS (3).

Increased level of genus *Streptococcus* has been previously described in Chinese and Japanese patients with MS, *Streptococcus* abundance negatively correlated with the proportion of T regulatory cells and positively correlated with Th17 cells in the peripheral blood (41). On the other hand, the anti-inflammatory activity of common human commensal *Streptococcus salivarius* K12 is attributed to its ability to avoid triggering an inflammatory response, achieved through inhibition of the NF-κB pathway, enhancement of type I and II interferon response, and modulation of genes associated with adhesion to the epithelial layer and homeostasis (42).

When analyzing patient groups according to their DMT, we again observed a decrease in recognized or putative SFCAs-producing bacteria in the non-responders’ groups. This included taxa *Coprococcus* (NR INF and NR FIN groups) (43), Lachnospiraceae (NR FIN, NR CLA) (44), Eggerthellaceae (NR FIN) (45), Christensenellaceae R7 group (NR IFN) (46), *Intestinimonas* (NR IFN and NR FIN) (47), *Faecalibacterium* (NR CLA)*, Eubacterium siraeum* group (48) and *Victivallis* (NR FIN) (49).

To summarize our findings on the abundance of different bacterial taxa in the distinct patient groups based on treatment type, treatment responders showed only few significant differences from HC in microbiome composition. However, a significant decrease in many groups of SCFAs-producing bacteria was observed in patients with active MS. In some groups of SCFAs-producing bacteria, this decrease was shared by the CIS patient group and NR to all types of treatment. Thus, reduction in SCFAs producing bacteria was a common finding in active MS groups as compared to HC or responders to treatment. SCFAs exert an anti-inflammatory and immunoregulatory actions that may help counteract the autoimmune processes underlying MS. By promoting regulatory T cells, decreasing pro-inflammatory mediators through several mechanisms in immune and non-immune cells and supporting barrier integrity, SCFAs show promise as an adjunctive strategy in MS management. This observation was also supported by the quantification of serum SCFAs, where patients with active MS (CIS, NR) has significantly lower levels of SCFAs in comparison with HC while responders had SCFAs levels similar to HC. Next to the changes in SCFA-producing bacteria and SCFAs levels in serum another important microbiota-related pathways are discussed with MS pathogenesis. Circulating bile acid profiles are altered in MS and associate with neuroinflammation, brain/retinal atrophy and disability-worsening risk, and tauroursodeoxycholic acid (TUDCA) supplementation in progressive MS is safe with measurable immunologic and microbiome effects (50–52). Microbial cholesterol-to-coprostanol conversion is a major gut route of cholesterol disposal shaped by community composition; coprostanol-forming taxa associate with lower fecal/serum cholesterol and more favorable lipid profiles, with potential implications for immune-cell lipid signaling (53–55). Microbiota-regulated tryptophan metabolism (kynurenine/indole pathways) is consistently disturbed in MS, including pediatric-onset disease, and influences CNS autoimmunity and T-cell balance (56, 57). Integrating these axes with emerging metabolomics/microbiome approaches for disease staging, progression monitoring and DMT selection/monitoring may help explain inter-individual variability in treatment response (58, 59).

The intestinal barrier is the essential component of the microbiota-gut-brain axis. One of its functions is to prevent translocation of antigens and microbes from the gut lumen. Maintaining gut barrier integrity is crucial for proper function of other components of this axis, including the immune system (60, 61). To assess the gut barrier function in CIS and during different DMTs, we investigated several serum biomarkers related to the immune response associated with microbial translocation, and its regulations.

In our study we found significantly elevated levels of MBL and LBP in patients with active MS (CIS and NR) as compared to HC. However, no significant differences were observed in patients with stable disease (R group). These findings suggested the disruption of gut barrier integrity in MS patients with active disease resulting in microbial translocation followed by inflammatory response. In MS, MBL may contribute to complement system activation, which could result in demyelination, damage of oligodendrocytes and infiltration of macrophages to the CNS (62). Plasma levels of MBL have been found to be significantly increased in patients with MS, myasthenia gravis and Guillain-Barré syndrome compared to healthy controls. Moreover, MBL plasma levels have been significantly correlated with severity of these autoimmune neurological disorders (63). LBP is one of the key components of the innate immune response against bacteria. It binds to LPS on bacterial surfaces and interacts with CD14 (64). A small study investigating pretreatment level of LBP in patients with RRMS reported higher levels compared to controls. At 56 weeks of treatment with natalizumab, these mean values were significantly reduced compared to baseline values. This aligns with our observations of elevated MBL and LBP in patients with active, but not in those with stable MS. Interestingly, we found only slightly elevated level of CD14 in CIS patients as compared to HC. Significantly elevated levels of CD14 were found later in both, stable and active MS patients as compared to HC. It was described that increased serum CD14 correlated inversely with disease activity in relapsing MS patients (65). Our observation cannot support this finding since both responders and non-responders had elevated levels of CD14. Besides the results mentioned above, in our cohort SAA, but not Lipocalin-2 captured the low-grade inflammation associated with early diseases pathogenesis as well as established MS, irrespective to treatment response. Previously it was shown that SAA levels are elevated in relapsing-remitting MS and correlated with MRI activity, suggesting that systemic low-grade inflammation tracks CNS lesion formation (66–68). Mechanistically, SAA can interact with the blood–brain barrier and impair its function, activate microglia and astrocytes, and promote differentiation of pathogenic Th17 cells, thereby amplifying CNS inflammation and neurodegeneration (69, 70) The significantly elevated SAA we observed in CIS and MS (responders and non-responders) compared with healthy controls is therefore consistent with a state of persistent systemic immune activation that is already present at the earliest clinically recognizable stage and remains detectable under DMT, irrespective of treatment response status. The absence of significant differences in serum Lipocalin-2 between healthy controls, CIS, responders and non-responders in our cohort suggests that Lipocalin-2 is not a sensitive peripheral marker of the inflammatory processes captured by SAA in early and treated MS.

IL-18 is a member of IL-1 superfamily, which includes eleven cytokines involved in the initiation and regulation of inflammation associated with most CNS diseases. Both *in vitro* and *in vivo* studies have shown that IL-1 cytokines family and receptors are altered in Alzheimer Disease and MS. These alterations in production of cytokines such as IL-1 and IL-18 likely switch their role from supporting neuroprotection and homeostasis to promoting pathological inflammation (71). Higher levels of IL-18 have been previously detected in the serum and cerebrospinal fluid (CSF) of patients with MS (72). Unlike our findings, other study reported significantly higher levels of IL-18 in patients with active MS than patients with inactive MS and HC (73). Significantly higher level of IL-18 in responders compared to CIS and NR, could suggest involvement of IL-18 in repairing processes of damaged tissue, while the disease may be non-active. Besides, it was shown that neuronal IL-18 directs killing of enteric bacterial pathogens and orchestrates mucosal barrier immunity by stimulating antimicrobial protein expression in goblet cells (74). We may only speculate, that high levels of IL-18 in the responders as compared to CIS and NR, could be the mechanism supporting mucosal barrier integrity and preventing the microbial translocation in these patients. Using the level of IL-18 we were able to distinguish responders from NR in both, IFN-β and FIN DMTs.

Calprotectin (S100A8/S100A9) belongs an important group of proteins of the innate immune system that promotes inflammatory responses (75, 76). Several studies have demonstrated significantly higher serum levels of calprotectin in patients with active compared to inactive MS. Serum calprotectin has been therefore proposed as a clinically useful biomarker of innate immune activation and disease activity (77, 78). In our study we found significantly higher levels of serum calprotectin only in CIS patients as compared to responders and NR. Therefore, it could not be used to distinguish treatment responses, even when patients were stratified based on specific DMTs.

Conversely, we observed significantly increased levels of OPN in both treatment responders and non-responders, but not in CIS. OPN is well known to bind to several integrin receptors, including α4β1 integrin, which is expressed on circulating T-cells and is essential for T cell homing in MS. In MS, microglia-derived OPN promotes the influx of lymphocytes, including T cells, increases their survival, but also regulates myelination and oligodendrogenesis (79, 80). A systematic review on studies measuring blood and CSF levels of OPN in MS patients and controls found that MS patients generally had significantly higher levels of OPN in both their CSF and blood when compared to all types of controls. All MS patients, except those with CIS, also exhibited elevated blood OPN levels compared to controls. When considering MS subtypes, individuals with CIS had significantly lower CSF OPN levels than RRMS patients and lower levels in both blood and CSF compared to patients with progressive subtypes of MS. Finally, patients with active MS had significantly higher OPN levels in their CSF compared to patients with stable disease (81). Increased OPN levels in our patients with a long disease duration (with median values of 6 years in IFN-β; 9.5 years in CLA and 16.5 years in FIN) compared to HC, but not in those at the very beginning of their disease, may indicate a distinct role of OPN in the early and advanced stages of MS. We did not find any significant differences between responders and NR. The levels of OPN were significantly higher in both, responders and NR when compared to HC. This could support the previous findings highlighting the role of OPN not only in inflammation but also in tissue repair (82).

## 5 Conclusions

In summary, we observed significant differences in the gut microbiota composition, both at the alpha and beta diversity levels, in individuals with CIS compared to HC. Upon treatment, only NR exhibited microbiota profiles similar to those of CIS patients, with no significant differences between responders and HC. When analyzing patients based on DMT type, no significant differences in alpha or beta diversity were found in responders and HC, except for those treated with CLA. At the taxonomic level, reduced levels of SCFA-producing bacteria were observed in CIS patients and NR compared to HC, which was supported with quantification of SCFA in serum. Additionally, we detected significantly higher levels of LBP and MBL in CIS patients and NR, indicating increased intestinal barrier permeability in active MS. In responders, a decrease in these biomarkers was observed. This finding can be interpreted in the context of SCFAs, which are anti-inflammatory and whose producers are notably more abundant in treatment responders than in NR.

## Data Availability

The datasets presented in this study can be found in online repositories. The names of the repository/repositories and accession number(s) can be found below: https://www.ebi.ac.uk/ena, PRJEB85230.

## Supplementary material

Supplementary Figure 1 Relative abundances of the top 18 most abundant taxa of gut microbiota at the genus level for every individual in each group.

Supplementary Figure 2 The levels of serum LBP, CD14, MBL, calprotectin, osteopontin and IL-18 in patients with CIS and RRMS patients according treatment and its response. Lipopolysaccharide-binding protein (LBP), soluble CD14 (CD14), Mannose-binding lectin (MBL), calprotectin, osteopontin, interleukin-18 (IL-18), Lipocalin-2, Serum Amyloid A (SAA). Statistical differences between groups were tested using the Kruskal-Wallis test with Dunn’s multiple comparisons *p<0.05, **p<0.01, ***p<0.001, ****p<0.0001.

## Glossary

AD: Alzheimer’s disease
ASVs: Amplicon Sequence Variants
BMI: body mass index
CD: cluster of definition
CIS: clinically isolated syndrome
CLA: cladribine
CNS: central nervous system
CSF: cerebrospinal fluid
CXCR: CXC chemokine receptor
DAMP: damage-associated molecular pattern proteins
DMT: disease modifying therapies
DNA: deoxyribonucleic acid
EAE: Experimental Autoimmune Encephalitis
EBV: Epstein-Barr virus
EDSS: Expanded Disability Status Scale
ELISA: enzyme-linked immunosorbent assay
ENA: European Nucleotide Archive
FIN: fingolimod
GALT: gut-associated lymphoid tissue
HC: healthy controls
HET: high-efficacy therapies
IFN: interferon
IL: interleukin
LBP: lipopolysaccharide-binding protein
LPS: lipopolysaccharide
MBL: mannose-binding lectin
MRI: magnetic resonance imaging
MS: multiple sclerosis
NA: not applicable
NEDA: No Evidence of Disease Activity
NF-κB: Nuclear factor kappa-light-chain-enhancer of activated B cells
NR: non-responders
NR group: non-responders
NR-CLA: non-responders to cladribine
NR-FIN: non-responders to fingolimod
NR-IFN: non-responders to IFN-β
OPN: osteopontin
PCoA: Principal Coordinate Analysis
PCR: polymerase chain reaction
PERMANOVA: Permutational Multivariate Analysis of Variance Using Distance Matrices
R: responders
R group: responders
R-CLA: responders to cladribine
R-FIN: responders to fingolimod
R-IFN: responders to IFN-β
RRMS: relapsing-remitting multiple sclerosis
rRNA: ribosomal ribonucleic acid
SAA: Serum Amyloid A
S1P: sphingosin-1-phosphate
SCFAs: short-chain fatty acids
SFB: segmented filamentous bacteria
SPMS: secondary progressive multiple sclerosis
Th: T helper cells
Tregs: T regulatory cells
