# Supplementary material for: The gut microbiota composition is shaped by disease activity and individual treatment responses in patients with multiple sclerosis
Source: Front Immunol. 2026 Feb 12;16:1681342. doi: 10.3389/fimmu.2025.1681342 (PMC12935932; doi:10.3389/fimmu.2025.1681342)
Supplement: Supplementary Figure 1 — Relative abundances of the top 18 most abundant taxa of gut microbiota at the genus level for every individual in each group. [file DataSheet1.docx]

## *Supplementary results*

*
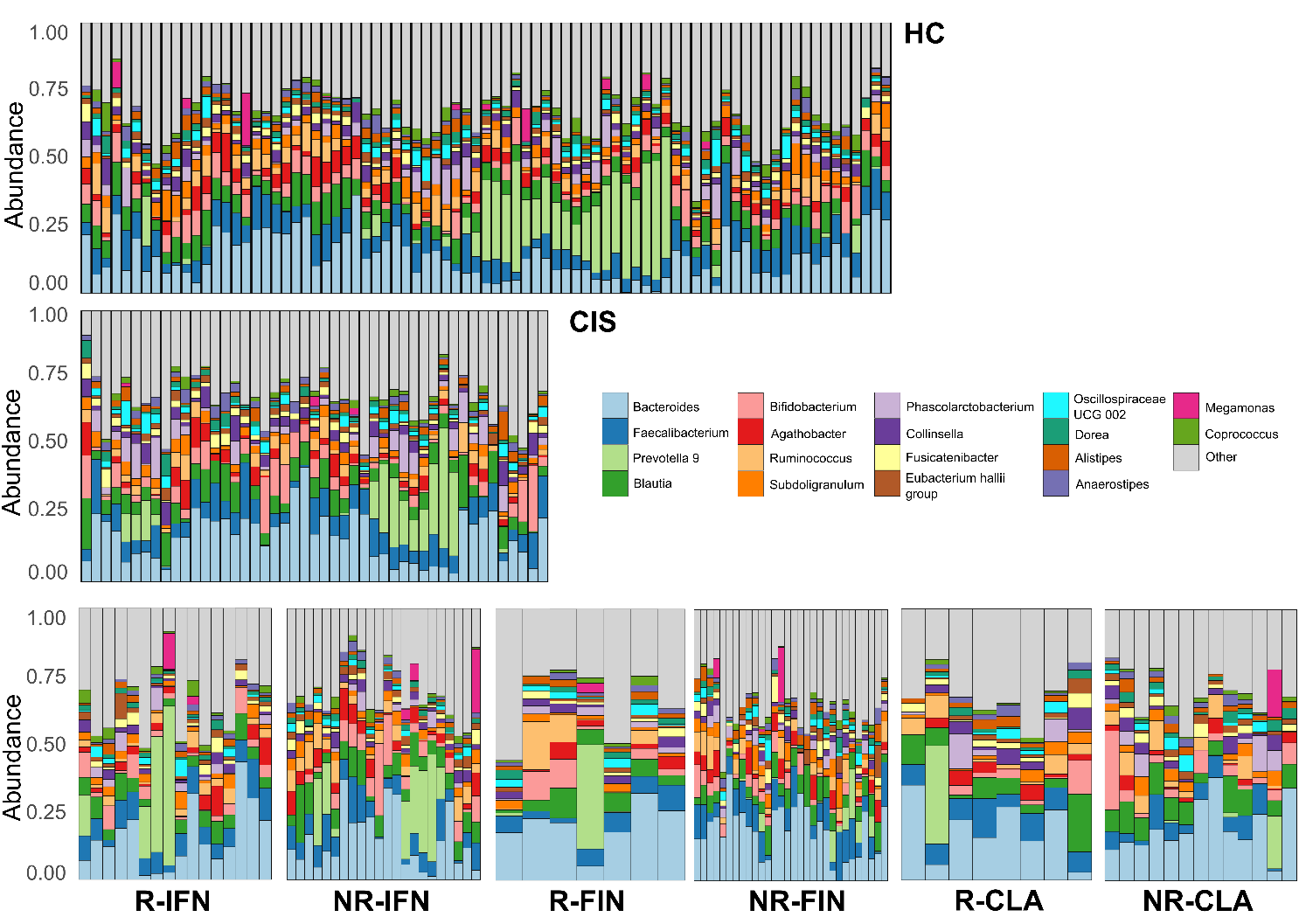
*

***Supplementary figure 1.*** *Relative abundances of the top 18 most abundant taxa of gut microbiota at the genus level for every individual in each group.*

##

***
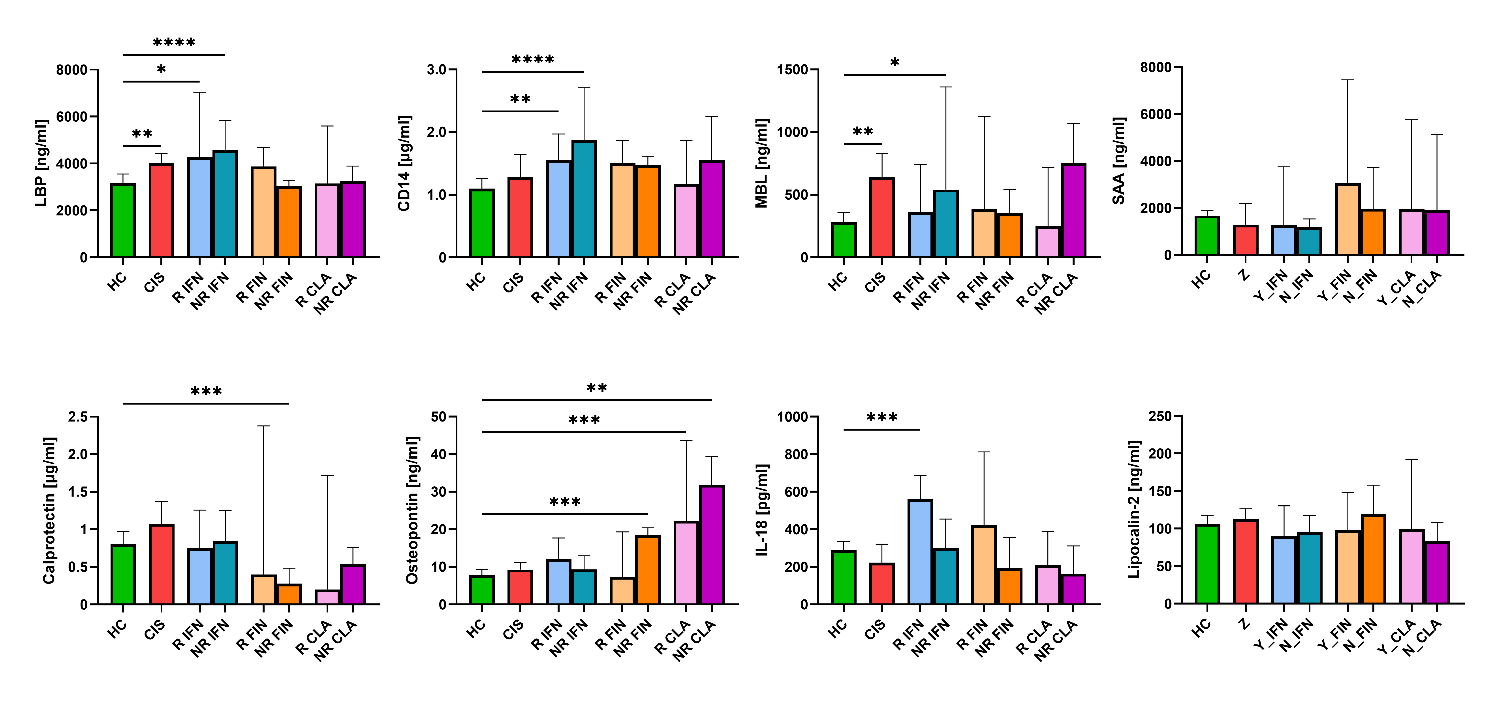
***

***Supplementary figure 2. The levels of serum in patients with CIS and MS patients with individual treatment response.*** *Lipopolysaccharide-binding protein (LBP), soluble CD14 (CD14), Mannose-binding lectin (MBL), calprotectin, osteopontin, interleukin-18 (IL-18), serum amyloid A (SAA), Lipocalin-2. Statistical differences between groups were tested using the Kruskal-Wallis test with Dunn’s multiple comparisons, *p<0.05, **p<0.01, ***p<0.001, ****p<0.0001 (HC n=63, CIS n=35, R-IFN n=16, NR-IFN n=21, R-FIN n=6, NR-FIN n=16, R-CLA n=8, NR-CLA n=5).*
